# Interest in an Online Smoking Cessation Program and Effective Recruitment Strategies: Results From Project Quit

**DOI:** 10.2196/jmir.8.3.e14

**Published:** 2006-08-22

**Authors:** Jennifer B McClure, Sarah M Greene, Cheryl Wiese, Karin E Johnson, Gwen Alexander, Victor Strecher

**Affiliations:** ^3^University of MichiganAnn ArborMIUSA; ^2^Henry Ford Health SystemDetroitMIUSA; ^1^Group Health Cooperative Center for Health StudiesSeatleWAUSA

**Keywords:** Internet, tobacco dependence, nicotine dependence, smoking cessation, recruitment activities

## Abstract

**Background:**

The Internet is a promising venue for delivering smoking cessation treatment, either as a stand-alone program or as an adjunct to pharmacotherapy. However, there is little data to indicate what percent of smokers are interested in receiving online smoking cessation services or how best to recruit smokers to Internet-based programs.

**Objective:**

Using a defined recruitment sample, this study aimed to identify the percentage of smokers who expressed interest in or enrolled in Project Quit, a tailored, online, cognitive-behavioral support program offered with adjunctive nicotine replacement therapy patches. In addition, we examined the effectiveness of several individual-level versus population-level recruitment strategies.

**Methods:**

Members from two large health care organizations in the United States were invited to participate in Project Quit. Recruitment efforts included proactive invitation letters mailed to 34533 likely smokers and reactive population-level study advertisements targeted to all health plan members (> 560000 adults, including an estimated 98000 smokers across both health care organizations).

**Results:**

An estimated 1.6% and 2.5% of adult smokers from each health care organization enrolled in Project Quit. Among likely smokers who received proactive study invitations, 7% visited the Project Quit website (n = 2260) and 4% (n = 1273) were eligible and enrolled. Response rates were similar across sites, despite using different sources to assemble the invitation mailing list. Proactive individual-level recruitment was more effective than other forms of recruitment, accounting for 69% of website visitors and 68% of enrollees.

**Conclusions:**

Smokers were interested in receiving online smoking cessation support, even though they had access to other forms of treatment through their health insurance. Uptake rates for this program were comparable to those seen when smokers are advised to quit and are referred to other forms of smoking cessation treatment. In this sample, proactive mailings were the best method for recruiting smokers to Project Quit.

## Introduction

In recent years there has been an explosive growth of Internet users around the world and a corresponding upsurge in interest in using the Internet to deliver online public health interventions such as smoking cessation treatment. The potential advantages of Internet-based treatment are clear. From the users' perspective, online treatment programs are convenient; content can be accessed 24 hours a day, 7 days a week, 365 days a year. They also offer a greater level of anonymity than in-person or phone-based counseling, which users may find appealing. From a delivery perspective, Internet programs allow rapid, broad, and economical treatment dissemination. Programs can be highly tailored to mimic the individualization of one-to-one counseling, and the Internet has the potential to reach audiences who might not seek services otherwise due to issues of cost, accessibility, or stigma.

Whether Internet-based smoking cessation programs will be as effective as person-to-person counseling remains to be proven. To date, very few randomized efficacy trials have been conducted [1], but some promising preliminary data [2-4] suggest that well-designed online cessation programs could be effective public health interventions, particularly when combined with pharmacotherapy [5].

The ultimate impact of any public health intervention, however, is dependent on its reach, as well as its efficacy [6]. Internet-based programs have the potential to reach millions of people, but potential reach is not actual reach. Actual reach requires access, acceptability, and utilization. While ongoing research seeks to establish the efficacy of online treatment, it is equally important to evaluate the acceptability and utilization of these programs in their target audiences. This assessment is hard to do because it requires a defined recruitment population and control over individuals' exposure to program advertisements, which is not possible in most research settings. No published studies to date, that we are aware of, have recruited smokers for Internet-based cessation treatment using a well-defined population that would allow accurate estimates of treatment uptake among smokers. Our best estimates come from surveys of Internet users. According to a recent Pew survey, 7% of adult US Internet users, approximately 8 million people, reported that they have searched online for information on how to quit smoking [7], but searching for information online is not the same as enrolling in an online cessation program. Joining a program requires a higher level of commitment and effort. This could partly explain why only 5-14% of smokers follow through with treatment referrals after being advised to quit [8-10] and less than 7% of smokers in the United States enroll in clinic-based cessation programs [11]. Research is needed that will allow us to better understand the acceptability and reach of Internet-based smoking cessation treatment. Moreover, it is important to understand how best to advertise these programs to smokers to maximize treatment uptake.

In this paper we report on smokers' interest in Project Quit, an online, individually tailored, cognitive-behavioral support program with adjunctive nicotine replacement therapy (NRT) patches. Participants were recruited from two large health care organizations in the United States using a combination of individual-level and population-level recruitment strategies. Working within the health care organizations provided a defined patient population, making it possible to estimate interest in this program among likely smokers who were invited to participate and to evaluate the effectiveness of our recruitment strategies.

## Methods

### Setting

Project 	Quit is a collaborative study between the University of Michigan 	(UM), Group Health Cooperative (GHC), and the Henry Ford Health 	System (HFHS). The primary purpose of Project Quit is to evaluate the "active ingredients" of an 	individually tailored, online smoking cessation program. A 	secondary aim is to evaluate smokers' interest 	in Web-based cessation treatment and evaluate optimal strategies 	for promoting this service among smokers. Project Quit is being 	conducted in two independent phases, each testing slightly 	different treatment content. This paper reports the recruitment 	outcomes for the first phase.

The 	Project Quit Internet program was primarily designed and maintained 	by the Center for Health Communications Research at UM. Study 	participants were recruited from the memberships of GHC and the 	Health Alliance Plan (HAP) of HFHS. Both GHC and HFHS are 	not-for-profit integrated health care delivery systems. At the time 	of this study, GHC served more than 540000 enrollees (adults and 	children) in Washington State and Idaho. An estimated 200000 adults 	and children in the greater Detroit, Michigan area were insured 	through HAP and received services through HFHS. Both GHC and 	HFHS/HAP provide behavioral counseling and pharmacotherapy for 	smoking cessation as covered insurance benefits, but at the time of 	this study neither offered an online cessation program.

All 	participants in this study received access to a tailored, 	cognitive-behavioral treatment program for smoking cessation that 	was delivered via the Internet. Treatment varied by the type and 	intensity of tailoring, but all participants received a personally 	tailored program and a 10-week supply of NRT patches. All treatment 	was provided free of charge. The study protocol was reviewed and 	approved by the Institutional Review Board (IRB) of each 	collaborating institution.

### Recruitment

 	Participants were recruited through a combination of 	individual-level and population-level strategies. Each of the two 	health care organizations identified likely current smokers via 	either automated smoking status data collected during recent 	medical appointments (Organization 1) or documentation of smoking 	in electronic medical charts, use of an internal list of smokers 	collected during prior research, or lists of patients with 	smoking-related conditions who had previously been prescribed 	cessation medications (Organization 2). Thus, all invitees were 	known to have been recent smokers with a high probability of 	current smoking. Likely smokers were prescreened for minimal 	inclusion criteria (eg, age) and were mailed a study invitation 	letter. The letter content was comparable across both health care 	organizations, but not identical due to different IRB requirements. 	Both letters briefly described the Project Quit program and study 	eligibility criteria and invited smokers to visit the Project Quit 	website to learn more about the study and be screened for 	eligibility. Individuals could also inform study staff if they did 	not want to be contacted further about this research. Finally, each 	site allowed people to refer friends and family members to the 	program, as long as referred smokers were members of one of the 	health care organizations. Information on how to refer a friend or 	family member was included in the invitation letter.

After 	approximately three months, we determined that we needed to boost 	our monthly enrollment rate to reach our recruitment goal during 	the study time frame. In an effort to expedite progress toward our 	overall recruitment goal, we amended the protocol to include a 	reminder mailing to likely smokers. Reminders were sent to all 	individuals who, at that point, had not yet visited the website or 	opted out of further contact regarding the study. From that point 	forward, reminder letters were sent to all persons who, four weeks 	after they received the initial invitation letter, had not visited 	the website or opted out of contact.

We also 	utilized several population-level enrollment strategies. The study 	was advertised in each health care 	organization's quarterly membership newsletter 	and was the focus of a feature article in one newsletter issue at 	Organization 2. Ads appeared in three to four issues total, 	depending on the site. Each site also advertised through a variety 	of supplemental strategies. Organization 1 highlighted the program 	in one issue of its staff newsletter and on the 	"Join a Study" page of the 	institution's website. Organization 2 advertised 	the study during a local promotion of the 2004 Great American 	Smokeout and allowed physician and nurse referrals, though the 	latter was not widely promoted among staff. Participants were 	actively recruited from September 2004 to July 2005.

Letters 	were proactively mailed to 34533 likely smokers at Organization 1 	(n = 18668) and Organization 2 (n = 15865). Quarterly newsletters 	were mailed to the entire membership of each health care 	organization, including approximately 563200 adults with GHC or HAP 	insurance coverage. Based on smoking prevalence data from automated 	medical records at Organization 1 and regional smoking prevalence 	estimates for Organization 2 [12], approximately 63180 adults at 	Organization 1 and 34506 adults at Organization 2 were smokers. At 	Organization 1, the staff newsletter ad was distributed to 	approximately 10000 employees, of whom 1000 were estimated to have 	been smokers based on internal smoking prevalence data among staff. 	It is not possible to estimate how many smokers were exposed to the 	other referral sources (eg, friend and family referrals, website 	posting).

Each 	recruitment strategy was associated with a unique referral code. 	Potential participants used these codes to log in to the Project 	Quit website. It is possible that some participants were exposed to 	more than one recruitment strategy (eg, invitation letter and 	newsletter ad); however, by using the referral codes we were able 	to track which promotional strategy they were responding to when 	they enrolled and to which health care organization they belonged. 	After logging into the site, individuals were able to read an 	overview of the study, be screened for eligibility, and provide 	informed consent.

### Participants

 	Individuals were eligible to participate if (1) they had smoked at 	least 100 cigarettes in their lifetime, currently smoked at least 	10 cigarettes per day, and had smoked in the past 7 days; (2) were 	seriously considering quitting in the next 30 days; (3) were 21 to 	70 years old; (4) were a member of GHC or HFHS/HAP; (5) had home or 	work access to the Internet and an email account that they used at 	least twice weekly; (6) were not currently enrolled in another 	formal smoking cessation program or currently using pharmacotherapy 	for smoking cessation; and (7) had no medical contraindications for 	NRT.

## Results

### Project Quit Recruitment Response

During 	the 11-month recruitment period for phase one of Project Quit, 3256 	people from both health care organizations visited the website; 	2651 were screened for eligibility (81% of website visitors); 2011 	were eligible (62% of website visitors); and 1866 enrolled (57% of 	website visitors).

We 	examined the response to each recruitment strategy by evaluating 	the number of people who responded to each and either visited the 	website to learn about Project Quit or consented and enrolled in 	the study (Table 1). Because the total response rate to each of the 	supplemental strategies (eg, friend and family referrals, website 	posting, staff newsletter, physician referral) was low, these 	strategies are combined into a single category in Table 1. Nearly 	9% of study participants (n = 159) were referred by friends or 	family, but response to each of the other supplemental referral 	sources ranged from 2 to 18 enrollees.

**Table 1:** Response to each recruitment strategy by health care organization

	**Visited Project Quit Website (N = 3256)**			**Enrolled in Study (N = 1866)**		
**Organization**	**Letter** **n (%)**	**Newsletter** **n (%)**	Other^*^ n (%)	**Letter** **n (%)**	**Newsletter** **n (%)**	Other^*^ n (%)
1	1224 (75)	260 (16)	136 (8)	730 (74)	171 (17)	85 (9)
2	1036 (63)	439 (27)	162 (10)	543 (62)	241 (27)	96 (11)
Both	2260 (69)	699 (21)	298 (9)	1273 (68)	412 (22)	181 (10)

^*^Includes friend and family referrals, web posting, 	staff newsletter, physician referral, and Great American Smokeout 	promotion.

The 	results suggest that the proactive invitation letters were superior 	to our other recruitment methods, accounting for 69% of people who 	visited the website and 68% of all enrollees. This finding was 	consistent across both health care organizations. A greater 	percentage of the Organization 1 sample was recruited by letter, 	but the response rate to the proactive letters was nearly equal in 	both samples. At Organization 1, 6.6% of letter recipients visited 	the website and 3.9% enrolled. At Organization 2, 6.5% of letter 	recipients visited the website and 3.4% enrolled. Of those who 	enrolled, 870 did so after receiving their first invitation letter 	and 403 did so in response to the reminder letter.

### Interest in Project Quit

The 	estimated percentage of adult smokers at each health care 	organization who enrolled in Project Quit was 1.6% and 2.5%, 	respectively, for Organization 1 and 2. Although newsletter 	advertisements were mailed to the entire membership of each health 	plan, there is no guarantee that smokers saw the population-level 	advertisements. Thus, a more valid estimate of 	smokers' interest in this program is based on 	the sample who received proactive invitation letters (n = 34533). 	Using this defined sample, we can better estimate the percentage of 	likely smokers who were interested in the online treatment program 	after learning about it: 7% of people who received a study 	invitation letter visited the Project Quit website (n = 2260), 6% 	of invitees were screened and eligible (n = 2011), and 4% of the 	total invitees (n = 1273), or 63% of those eligible, enrolled.

In 	total, 651 people were found to be ineligible for this study. The 	primary reasons for ineligibility were not smoking enough (26%), 	medical contraindications for NRT (23%), already being enrolled in 	another smoking cessation program (16%), lack of adequate 	Internet/email access (14%), not currently being enrolled in a 	participating health plan (10%), and currently using 	pharmacotherapy to quit smoking (8%). Of those who were ineligible, 	462 visited the website in response to an invitation letter. 	Compared to persons recruited through all other methods (n = 189), 	invitation letter recipients were less likely to be ineligible due 	to age (0.2% vs 2.6%, *P* = .03) or not being currently 	enrolled in a participating health plan (3.9% vs 25.9%, *P* < .001) and more likely to be ineligible due to current use of 	another smoking cessation program (11.7% vs 4.2%, *P* = .003) 	or a medical contraindication for NRT use (26.0% vs 15.3%, *P* 	= .003). These differences are consistent with our methods for 	identifying letter recipients.

### Enrolled Participants

The 	demographic characteristics of enrolled participants are presented 	in Table 2. The sample is similar to smokers who enroll in phone 	counseling programs in that they were ready to quit and were 	middle-aged, moderate-to-heavy smokers with a history of numerous 	quit attempts [13-15]. The subsamples differed slightly by health 	care organization; Organization 2 participants were less likely to 	be married or living with a partner (*P* < .001), less 	educated (*P* < .001), less likely to be White (*P* < .001), less comfortable using the Internet (*P* = .02), 	and smoked slightly more cigarettes per day (*P* < 	.001).

**Table 2:** Characteristics of enrolled participants

	**All** **(n = 1866)**		**Organization 1** **(n = 986)**		**Organization 2** **(n = 880)**	
**Characteristic**	**n**	**%**	**n**	**%**	**n**	**%**
Female	1110	59.5	586	59.4	524	59.5
Married/living with partner	1278	68.5	682	69.1	595	67.6
Employed	1421	76.2	749	76.0	672	76.3
Education^*^						
High school/GED or less	451	24.2	204	20.6	246	28.0
Vocational/technicalschool	222	11.9	141	14.3	81	9.2
Some college	1050	56.3	564	57.0	486	55.2
Postgraduate degree	136	7.3	71	7.2	65	7.4
Caucasian^*^	1486	79.6	831	84.3	655	74.3
3 or more prior quit attempts^†^	1218	65.3	668	67.7	550	62.5
	**Mean**	**SD**	**Mean**	**SD**	**Mean**	**SD**
Age	46.3	10.7	46.5	11.1	46.1	10.2
Cigarettes per day^*^	21.8	9.3	21.0	8.6	22.7	9.9
Motivation to quit^‡^	8.3	1.7	8.3	1.7	8.3	1.8
Comfort using the Internet^† ,‡^	6.8	3.7	7.0	3.7	6.6	3.7

^*^Significant difference between organizations, *P* < .001

^†^Significant difference between 	organizations, *P* < .05

^‡^Scores range from 1 to 10.

We also 	compared participants who were recruited by proactive invitation 	letter to those recruited by newsletter. Newsletter recruitees were 	more likely to be female (64.1% vs 58.2%, *P* = .03), 	Caucasian (88.6% vs 77.5%, *P* = .06), and older (47.0 vs 45.0 	years, *P* = .001). There were no significant differences in 	education, marital status, motivation to quit smoking, comfort 	using the Internet, or the number of cigarettes smoked per day.

## Discussion

### Principle Results

We found that smokers were interested in participating in Project Quit, a Web-based smoking cessation treatment program, even when they had access to other forms of comprehensive intervention through their health insurance. Of those who received a study letter and were invited to be screened for eligibility, 7% visited the website and 4% were eligible and enrolled. While these numbers may appear low, they are comparable to follow-through rates (5-14%) for referrals to other forms of cessation counseling [8-10]. Moreover, nearly two-thirds of those eligible (63%) agreed to enroll.

To our knowledge, this is the first study to document the level of interest in an online smoking cessation treatment program. We believe this is an important finding. Online cessation programs are becoming more prevalent on the Web. Whether or not they will be as efficacious as person-to-person counseling remains to be proven, yet no matter how efficacious an Internet cessation program is, its effectiveness will ultimately be dependent on its acceptability and utilization. These findings suggest that online cessation treatment can have comparable appeal to other forms of behavioral counseling, especially when part of a comprehensive intervention that combines cognitive behavioral counseling with pharmacotherapy, as is the best practice recommendation for tobacco dependence treatment [11].

While the uptake rate for Project Quit is comparable to that of other forms of therapy, these results may not generalize to other online cessation programs. Based on participants' self-report at follow-up, we know that a substantial portion of smokers were interested in receiving NRT. Online programs that do not offer the option of pharmacotherapy may be less appealing to smokers, at least to those with adequate health care coverage and other treatment options. Furthermore, our enrollment rate may have been limited by the eligibility criteria of our study. We selected adult smokers, with access to the Internet, who were ready to quit smoking and had no contraindications for NRT use. Higher enrollment may be seen for programs with less restrictive inclusion criteria. Finally, responses rates may differ in populations with different base rates of smoking. Our primary take rate (4%) is based on the percentage of likely smokers who received a proactive letter announcing the program. We selected people to receive these invitation letters based on internal data documenting their recent smoking. Unfortunately, population-level annual quit rates are fairly low in the United States. Each year, only about 2.5% of smokers successfully quit smoking permanently [16]. Thus, we have reasonable confidence that the majority of individuals contacted were still smoking when they received the letters, but we cannot confirm the exact percentage who were smoking at contact. Less treatment interest may be found in future populations if the base rate of smoking is lower than in this study, and vice versa.

As a secondary outcome we examined the success of our various recruitment strategies and found that proactive, individual outreach was a more effective recruitment strategy than mass advertising. More study participants visited the website and enrolled in response to proactive invitation letters than to all other forms of recruitment. This finding may not be surprising. While our population-level advertisements had the potential to reach a greater number of people (> 560000 adults), there was no guarantee that they were actually seen by their intended audience of smokers (approximately 98000 adults). Consequently, we cannot directly compare the draw of the newsletter ads to our invitation letters or other referral strategies, but we can comment broadly on the effectiveness of each strategy as a means of outreach for this study. In addition, we cannot assume that people were not exposed to more than one recruitment strategy or that multiple exposures did not have some impact, but using our unique referral codes, we can state with confidence which promotional strategy participants were responding to when they visited the Project Quit website. Nearly 70% of all visitors responded to the invitation letters proactively mailed to likely smokers. This finding has implications for future research, as well as community-based treatment dissemination efforts. Proactive contact was possible in this trial because of our access to automated data and other internal indicators of smoking status, but a similar outreach strategy could be implemented in the community using commercially available mailing lists of smokers or mailing lists from state or national smoking quit lines of likely smokers. More widespread recruitment could be achieved via commercially available email address lists. Even if it were not possible to limit email distribution to likely smokers, the cost per recipient would be low enough to make this a cost-effective recruitment strategy.

### Conclusion

The results of this study add to the small but growing literature on Internet-based smoking cessation treatment and suggest that online intervention can be as appealing to smokers as other forms of treatment, but utilization may be dependent on the overall program content and effective promotional outreach. Future research should continue to evaluate smokers' interest in using online services, among both insured and uninsured individuals. Additional methods for promoting utilization of online programs should also be explored. A greater understanding of these issues will be important for effectively delivering efficacious online cessation services in the future.

## Abbreviations

GHC: Group Health Cooperative
HAP: Health Alliance Plan
HFHS: Henry Ford Health System
IRB: Institutional Review Board
NRT: nicotine replacement therapy
UM: University of Michigan
